# Report of Two Cases of Spinal Meningitis

**Published:** 1872-07-15

**Authors:** F. H. Jennings

**Affiliations:** Marshall, Clark County, Illinois


### REPORT OF TWO CASES OF SPINAL MENINGITIS.

*Read before the Clark County Medical Society, July 3d, 1872.*

BY DR. F. H. JENNINGS, JR., OF MARSHALL, CLARK COUNTY, ILLINOIS.

(*Ordered Printed by the Society.*)

*Case 1st.* A female child, aged seven years, previously hearty. Monday, May the 27th, looked pale and refused breakfast; went to bed; said she had pain in the head and back. Her stomach became irritable, which was soon followed by vomiting a bilious matter; her pulse became quick and irregular; temperature of the body, high; bleeding at the nose, pupils dilated, constipation of the bowels, and partial opisthotonos; these symptoms yielding to the following treatment:

Purgatives: Calomel, quinine and iron, blistering over the region of the spine, sinapisms to the inferior extremities, and cold to the head.

On June the 9th she relapsed by exposure and neglect; symptoms assumed a more grave character: Strabismus of right eye, partial paralysis of right side, confused memory, loss of speech, and involuntary evacuations from the bowels, urine high color, but no distended bladder, constipation of the bowels, hypersæthesia, and convulsions.

Treatment: Blistering to the spine, sinapisms to the extremities, and cold to the head.

℞ Iodide Potassa, } a. a. ʒ ss.

Bromide " }

Aqua, Fontana, f ℥ ij. m.

Dose: One teaspoonful every four hours. Continued recipe three days, to no favorable result; ordered discontinued.

℞ Hyd. Sub. Mur. gr. ss.

Pulvis Opii gr. ¼

Pulvis Camphora, gr. ss. m.

S. One powder every three hours, with the addition of solution of strychnia; symptoms better.

By the advice of my esteemed friend, Dr. F. R. Payne, solution of strychnia discontinued and substituted elixir Ferri, calisaya and strychnia. She continued to improve slowly and was taking nourishment. By the protractedness and tediousness of the case, and the interference of meddlesome persons destitute of manly principles, she fell from my hands. While penning these lines, I learn from the parents that she relapsed the second time, and is still having those horrible convulsions almost daily, and that the attending physician gives it as his opinion that it is rotten worms in the bowels, and that she has no symptoms of spinal meningitis and never had.

*Case 2d.* Was a male child aged five years, previously healthy. June 1st was taken with pains in the head, stiffness of the neck, pain in the back, vomiting; temperature of the body high, tongue coated with brown fur, and edges red, pupils dilated, constipation of the bowels, pulse quick and regular.

Treatment: Purgatives—calomel quinine and iron. Blistering to the spine, sinapisms to the extremities, and cold to the head, under which treatment he improved, but afterwards relapsed under the same circumstances as case No. 1. Symptoms then assumed a more grave character, complete opisthotonos, dilated pupils, pain in the head and back, vomiting, and diarrhœa, with distended bladder, and a purple eruption extending over the body, which lasted for two days.

℞ Hyd. Sub. Mur. gr. ss.

Pulv. Ip. et Opii gr. ij.

Pulv. Camphora gr. ss. m.

S. One powder every three hours, with the addition of elixir ferri calisaya and strychnia. As a diuretic, I ordered spirits nitre, dulc. gtts. 10 every hour.

Local treatment: Blistering to the spine and cold to the head, under which treatment he continued to improve, but I lost the opportunity of witnessing the realization of my most sanguine expectations as the result of the foregoing treatment by the same intermeddling persons as in case No. 1.

